# The Cytoskeletal Structure in Cardiomyocyte Maturation and Proliferation

**DOI:** 10.3390/cells14191494

**Published:** 2025-09-24

**Authors:** Aldana Rojas, Shelby Dahlen, Feng Zhang, Shijie Liu

**Affiliations:** 1 Division of Molecular Cardiovascular Biology, Cincinnati Children’s Hospital Medical Center, Cincinnati, OH 45229, USA; aldana.rojas@cchmc.org (A.R.); shelby.dahlen@cchmc.org (S.D.); feng.zhang@cchmc.org (F.Z.); 2 Department of Pediatrics, University of Cincinnati, Cincinnati, OH 45229, USA

**Keywords:** cardiomyocyte maturation, dedifferentiation, proliferation, cytoskeleton, metabolism

## Abstract

The adult heart has a limited ability to regenerate, which is partly due to the structural and metabolic specialization that cardiomyocytes (CMs) acquire during postnatal maturation. In this review, we explore how cytoskeletal remodeling, metabolic reprogramming, and interactions with the extracellular matrix (ECM) regulate CM maturation, proliferation, and the potential for regeneration. We describe how the assembly of microtubules, actin filaments, and sarcomeric structures is essential for developing contractile function, but also creates structural barriers that prevent cell division. Recent studies show that disassembling these cytoskeletal components, along with activating signaling pathways such as Hippo-YAP, Wnt, and NRG1/ErbB4, can promote CM dedifferentiation and re-entry into the cell cycle. Metabolic shifts also play a critical role. A return from oxidative phosphorylation to glycolysis also leads to CM dedifferentiation and proliferation. In addition, changes in ECM composition and mechanical signaling affect cytoskeletal dynamics and regenerative capacity. Understanding how these structural, metabolic, and signaling networks work together opens the door to new approaches for restoring heart function after injury.

## 1. Introduction

Cardiomyocytes (CMs) are the basic beating units of the heart, which provide the contractile force that pumps blood around the cardiovascular system. During heart development, CMs undergo several complex changes that allow them to sustain lifelong contraction both mechanically and energetically [1,2]. They transition from a fetal structure, compatible with proliferation, to a mature, stiff, robust structure able to sustain repeated stress. These changes involve shifts in metabolism, sarcomere organization, and centrosome disassembly that prevent future proliferation once the cell reaches maturation [2,3]. This trade-off in the cell prevents the heart from repairing itself after cardiac injury.

The cytoskeleton plays a principal role in CM maturation and proliferation. Actin filaments, microtubules, and intermediate filaments provide structural stability, organize cellular components, and aid in signal transmission and cell shape [4]. As the cell matures, these structures rearrange and give rise to the sarcomere, essential for heart contraction [1,4]. The sarcomere is the most prominent structure in mature cardiomyocytes and provides contractile force to the cell, while the other components of the cytoskeleton distribute these mechanical forces and keep organelles in place [1,4]. During cardiac injury, some CMs undergo changes that allow them to revert to a proliferative stage, adapting the cytoskeleton to be able to re-enter the cell cycle [5,6].

In this review, we explore how the cytoskeleton influences cardiac maturation and proliferation and its changes during CM dedifferentiation. We begin by highlighting the key features of cardiac cell maturation and describe how the cytoskeleton adapts to support the structural and functional needs of the cell [3,7]. Next, we investigate the molecular mechanisms of CM proliferation and how the cytoskeleton contributes to the dedifferentiation process along with cell cycle progression and metabolic changes [2,8,9,10,11]. We also describe cytoskeletal interactions with the extracellular matrix and how they influence the cell’s regenerative potential [8,12,13]. Finally, we dedicate a section to the robust sarcomeric cytoskeleton, its reorganization during cell cycle re-entry, and its role in contraction. By bringing together current research, we aim to provide new perspectives on the importance of targeting the cytoskeleton and metabolism to unlock the heart’s regenerative capabilities.

## 2. Cardiomyocyte Maturation

### 2.1. Physiological Maturation

**Figure 1:**
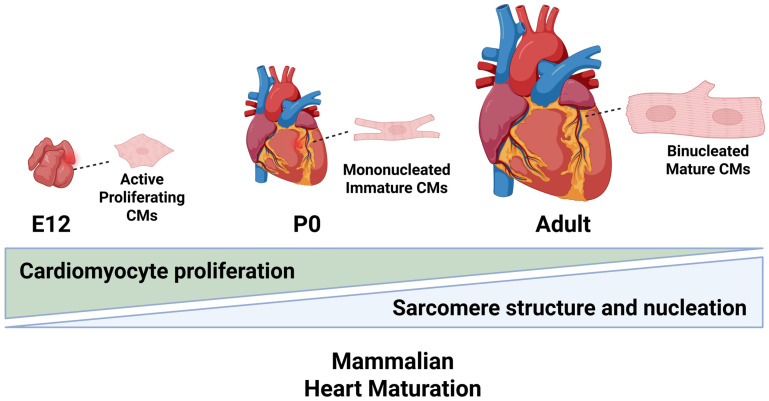
Stages of mammalian heart and CM maturation. During embryonic development (E12), the heart is primarily composed of actively proliferating cardiomyocytes. At birth (P0), the heart contains mononucleated CMs and functional remodeling begins. In the adult heart, cells have exited the cell cycle and become fully mature and non-proliferative, exhibiting aligned sarcomeres, nucleation, and increased cell size. Created in BioRender.

Mammalian heart development depends entirely on the proper cardiomyocyte proliferation and maturation processes that will allow the organ to grow and meet its structural and metabolic needs [7]. Once cardiomyocytes reach maturity, they exit the cell cycle and are unable to divide [14,15]. This process ensures that the cytoskeleton and the tissue remain strong to allow for lifelong contraction and blood circulation. As illustrated in Figure 1, in mice, most CMs exit the cell cycle during the first week after birth. By postnatal day 14 (P14), about 90% of the CMs are binucleated [16,17]. This increase in binucleation is a result of the cell undergoing DNA replication without proper mitosis or cytokinesis. On the other hand, human CMs are mostly mononucleated and instead display increased polyploidization [18,19,20].

As previously mentioned, CM maturation involves several changes that affect metabolism and the cytoskeleton. On the metabolic side, CMs experience an increment in both size and number of mitochondria to increase the amount of energy produced to sustain tissue contraction [18,21]. Meanwhile, the cytoskeleton favors an increment in contractile force and changes in the plasma membrane that improve the propagation of action potentials and excitation-contraction coupling [22,23]. All of these changes are accompanied by changes to gene expression and biochemical signaling that give rise to the fully electrophysiologically and metabolically developed CM.

Additionally, CM maturation has an impact on cell size. Cytoskeletal remodeling is largely responsible for the changes in cell size and shape [24,25]. The cytoskeleton also plays a central role in regulating cellular physiology through functions such as determining cell polarity and delivering mechanical and biochemical signals. Cell polarity is not only essential for coordinating tissue-level function but also serves as a major regulator of the Hippo pathway, as apical–basal polarity complexes (Crumbs, aPKC, Scribble) directly interact with Hippo components to control YAP/TAZ localization and activity [26]. The cytoskeleton is able to integrate intracellular signaling with external mechanical cues, ensuring neighboring CMs are properly coordinated at the tissue-level function [27,28].

### 2.2. Myofibril Maturation

**Figure 2:**
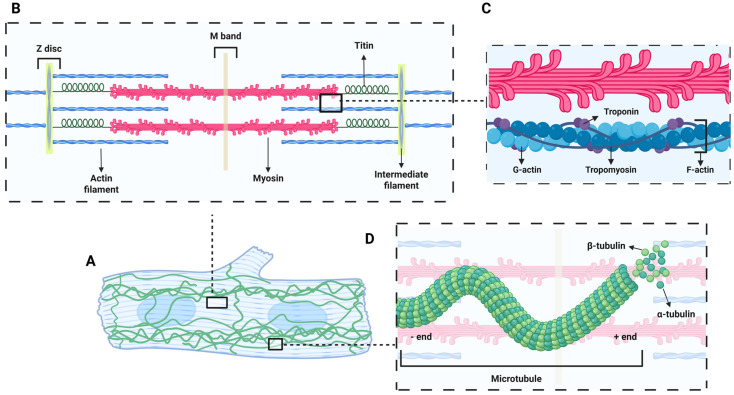
Structural organization of the CM cytoskeleton. (**A**) A mature CM, highlighting the integration of sarcomeres, actin filaments, and the microtubule network. (**B**) The sarcomere structure, with key components including actin (thin) filaments anchored at the Z-disc, myosin (thick) filaments across the M-band, titin connecting Z-discs to the M-line, and intermediate filaments contributing to structural integrity. (**C**) The actin filament, showing G-actin monomers polymerizing into F-actin, which is stabilized by tropomyosin and regulated by troponin. (**D**) The microtubule network composed of α- and β-tubulin heterodimers, with dynamic + and − ends, contributing to intracellular transport and mechanical stiffness. Created in BioRender.

As demonstrated in Figure 2, the sarcomeric cytoskeleton, composed of sarcomeres organized into myofibrils, is anchored by Z-discs and contains myosin, actin, troponin, and tropomyosin [29,30]. As CMs mature, myofibril density increases to allow for cardiac growth and contraction performance. Sarcomeres also influence gene expression through the ACTIN-MRTF-SRF pathway [1]. The serum response factor (SRF) is an important transcription factor for CM maturation since it promotes the expression of sarcomere-associated genes, which are involved in a feedback loop that supports cytoskeletal development and heart growth [1].

As the cell begins to exit the cell cycle and reaches maturation, sarcomere assembly is a prerequisite for cardiomyocyte specification, while myofibril expansion marks a hallmark in functional maturation [1,31]. Transcriptional control and alternative splicing are responsible for the transition from fetal sarcomeric isoforms to adult variants that will sustain a robust myofibril development [32,33,34].

In mice, myosin heavy chain expression changes from the fetal isoform MYH7 to the mature MYH6. In contrast, MYH7 governs the human adult heart starting from the fifth week of gestation [35]. At the same time, fetal myosin light chain is expressed as MYL7 (also known as MLC-2a) and MYL2 (MLC-2v) in ventricular CMs, while MYL7 expression becomes limited to atrial CMs. Moreover, fetal cardiomyocytes express slow skeletal troponin I (TNNI1) that gets replaced later by cardiac troponin I (TNNI3) as the cells mature [36].

Even though an increase in myofibrils marks a before-and-after in cardiac maturation by allowing proper sarcomere assembly, most of the molecular mechanisms behind sarcomere and myofibril expansion remain unclear.

### 2.3. Non-Sarcomeric Cytoskeletal Maturation

The non-sarcomeric cytoskeleton is also important during cardiac maturation. Elements like tubulin, actin, and desmin form microtubules, F-actin filaments, and intermediate filaments, respectively [37]. Microtubules are composed of alpha and beta tubulin and form highly dynamic structures that polymerize and depolymerize depending on the cell needs. While most of the tubulin remains free in the cytoplasm, the ones that compose the microtubular structure are critical for assembling the mitotic spindle and ensuring accurate genetic information segregation during cell division [38,39]. If the microtubule network is disrupted, the spindle apparatus cannot attach to the kinetochores in the chromosomes, and cell division is impaired [40,41]. Moreover, this dynamic characteristic of the microtubules allows the cell to adapt to mechanical forces by adjusting the cell size and shape [42,43,44]. Microtubule density peaks during early postnatal development and quickly decreases as CMs mature [45]. This is accompanied by a reduction in overall tubulin and polymerization levels, illustrating how microtubules adapt to the new demands of a contractile heart. How these changes intersect with proliferative signals remains mostly undiscovered.

Microtubules interact closely with other parts of the cytoskeleton, such as the actin and desmin networks, to regulate myofibril assembly and maintain sarcomere stability [46].

Desmin, the main intermediate filament in cardiac muscle, maintains structural integrity and organelle organization, and its misfolding or aggregation contributes to heart failure [47,48]. While transient Desmin aggregation may buffer mechanical and redox stress, chronic accumulation disrupts proteostasis and drives disease [49]. Early and sustained Desmin expression in embryonic stem-cell-derived cardiomyocytes enhances early cardiomyogenesis and drives the formation of large, highly interconnected clusters of synchronously contracting cardiomyocytes [50]. F-actin, primarily composed of a CM-specific isoform (ACTC1), pairs with α-actinin-2 (ACTN2) at the Z-discs to increase sarcomere organization and form the contractile ring for cytokinesis [51]. For this to occur, tight actin regulation is needed: overexpression of actin depolymerizing factor (ADF) arrests the cell cycle at G1, while excessive F-actin polymerization disrupts mitosis and cytokinesis, leading to multinucleation [52,53]. Cofilin acts an actin-depolymerizing factor that promotes myofibril maturation in response to mechanical stress during heart development by regulating how vinculin distributes and activating slingshot protein phosphatase 1 (SSH1), both key factors in cytoskeletal remodeling [54].

### 2.4. Maturation of Cell-Membrane-Associated Structures

Other structures, such as the intercalated discs, also help coordinate both remodeling and function. Intercalated discs connect neighboring CMs and facilitate mechanical and electrical coupling, essential for coordinated contraction [55]. The primary membrane-associated structure, the costamere, is composed of several complexes such as the dystrophin-glycoprotein complex (DGC) and the integrin–vinculin–talin complex, which transmit biochemical signals between the sarcomere and the ECM [56].

Intercalated discs physically connect neighboring cardiomyocytes, enabling the mechanical and electrical coupling necessary for coordinated contraction. They are composed of numerous components organized in a highly structured manner, forming a complex network that links neighboring proteins and the cytoskeleton [55]. This intricate assembly of junctional proteins, ion channels, and connexins is referred to as the area composita. Proteins such as N-cadherin (N-CAD), β-catenin, and plakoglobin (JUP) are critical for mechanical coupling between cardiomyocytes [55].

During CM maturation, these intercalated disc proteins—as well as gap junction proteins like connexins—are initially distributed throughout the cell membrane. Over time, they gradually become restricted to the lateral surfaces and ultimately concentrate at the longitudinal ends of the cells [57,58]. Disrupted expression or incorrect localization of these proteins has been associated with cardiomyopathies and an increased risk of arrhythmias, underscoring the vital link between mechanical structure and electrical function in the heart [59,60]. All these structures demonstrate how a coordinated and delicate cytoskeletal balance is needed to determine the proliferative fate of cardiac cells.

### 2.5. Metabolic Maturation

An increase in ATP production is necessary for a mature cardiomyocyte to sustain the energetic needs of a contractile heart. The transition from glycolysis to oxidative phosphorylation allows for greater energy output, but it comes with a trade-off: an increase in the production of reactive oxygen species (ROS) that cause DNA damage and permanent exit from the cell cycle [61]. Succinate dehydrogenase (SDH) is an enzyme that oxidates succinate and boosts ROS levels during ischemia [62]. Pharmacological inhibition of SDH with malonate reduces ROS present and stimulates cell cycle re-entry and myocardial regeneration after injury [62]. In mature CMs, mitochondria occupy up to 40% of the cell’s total volume. It is no surprise then that the mitochondrial network is highly interactive with the cytoskeleton [7].

Cytoskeletal dynamics not only affect cell cycle progression but also help in metabolic maturation. Regulation of oxidative phosphorylation in CMs depends, at least in part, on interactions between the mitochondria and cytoskeletal proteins [4]. For example, the actin cytoskeleton interacts with mitochondria by anchoring the organelles next to and providing transport to regions with a high-energy demand, while long range transport relies primarily on the microtubule network [63]. A recent study showed that microtubules facilitate the transport of mitochondria between assembling myofibrils through a conserved kinesin-dependent mechanism from flies to mice. Disruption of microtubules leads to immediate impairments in both mitochondrial organization and myofibril formation [64]. Moreover, sarcomere disassembly has been associated with a reduction in mitochondrial size, illustrating a close relationship between structural and metabolic remodeling during CM maturation [31].

### 2.6. Centrosome in Cardiomyocyte Maturation

Permanent cell cycle exit in cardiomyocytes requires the dismantling, suppression, and in some cases, physical relocation of the cellular replication machinery [3]. During CM maturation, the centrosome—which serves as the microtubule-organizing center (MTOC)—undergoes structural reorganization. Key components such as pericentriolar material 1 (PCM1) and pericentrin (PCNT) relocate from the centriole to the nuclear envelope in a process known as centrosome reduction. This involves the disassembly of pericentriolar proteins like CEP135 and a shift in isoforms from PCNT-B to PCNT-S, a molecular switch linked to cell cycle arrest [3]. These changes give rise to a new, nuclear-associated MTOC [3], making the nuclear envelope the dominant structure in adult CMs [65].

Centrosome reduction appears to be closely tied to CM maturation: inducing it pharmacologically promotes maturation, while impaired reduction delays it [3,66]. These findings suggest that microtubule nucleation is intimately linked to CM maturation, dedifferentiation, and potentially CM proliferation. Interestingly, this process is species-specific—for instance, zebrafish CMs retain centrosomal structure and remain capable of proliferation into adulthood [65].

It is important to note that disruptions in centrosome function have been linked to cardiac development disorders, including nonsyndromic dilated cardiomyopathy (DCM) [3]. For example, mutations in proteins critical for centriole assembly, like rotatin (RTTN), compromise centriole assembly and centrosome organization during CM maturation, which in turn impact microtubule organization, mitochondrial transport, fission, and fusion, influencing the CM maturation and cardiac function [3].

On the other hand, in some mature cells, the centrosome associates with the plasma membrane to form a primary cilium, a structure involved in mechano-and-chemosensing, motility, and signal transduction [67]. The primary cilium (also known as basal body) can regulate important signaling pathways such as Hippo, Wnt, and Notch, all of which act during cell proliferation and tissue development [68]. However, this cilia structure is temporary in CMs, seen firstly during early development and absent when cells reach maturation.

Altogether, these findings highlight the importance of the centrosome beyond mitosis alone and its role in CM metabolism and contractility regulation.

## 3. Cardiomyocyte Proliferation and Dedifferentiation

**Figure 3:**
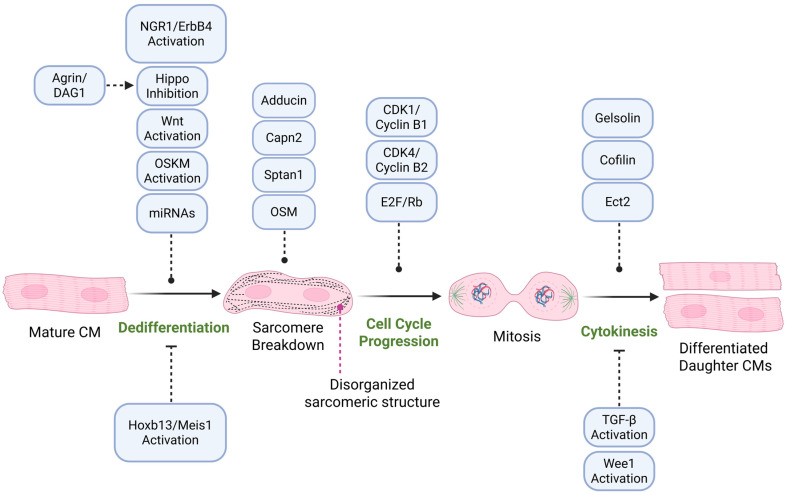
Overview of CM dedifferentiation, proliferation, and redifferentiation. The process begins with dedifferentiation of mature CMs, characterized by sarcomere disassembly and morphological remodeling, followed by cell cycle re-entry, mitosis, and eventual cytokinesis. Key regulatory pathways and molecules involved at each stage are shown. Dedifferentiation is promoted by Hippo inhibition, Wnt and OSKM activation, miRNAs, and NRG1/ErbB4 signaling, and may be enhanced by Agrin/DAG1. Dedifferentiation is blocked by Hoxb13 and Meis1. Sarcomere breakdown is driven by Capn2, Sptan1, Adducin and Oncostatin M (OSM). Cell cycle progression is regulated by CDK1/cyclin B1, CDK4/cyclin B2, and E2F/Rb signaling. Mitosis proceeds to cytokinesis, which is facilitated by actin-regulatory proteins such as gelsolin, cofilin, and Ect2, while being inhibited by TGF-β and Wee1 activation. The final step involves redifferentiation of daughter CMs into functional cardiac cells. Created in BioRender.

For many decades, the idea of a CM re-entering the cell cycle was considered impossible due to the vast epigenetic reprogramming the cells undergo during maturation. Chromatin remodeling, histone modifications, and DNA methylation, among others, silence genes that regulate cell cycle, metabolism, and the cytoskeleton, preventing cell proliferation [69]. However, recent evidence demonstrated that mature CMs can re-enter the cell cycle under certain conditions through a process of dedifferentiation that involves cytoskeletal remodeling and changes in metabolism that resemble those of the fetal stage [70,71,72]. The disassembly of the sarcomere, the breakdown of the actin cytoskeleton coupled with the reactivation of fetal genes, allow CMs to go back to a more proliferative state [72,73,74]. A synopsis of these mechanisms and the factors that mediate them is shown in Figure 3.

Mechanisms such as the Hippo pathway, as well as Wnt and NRG1/ErbB4 pathways allow for this reprogramming and are typically turned on or off by mechanical or chemical signals detected by the cytoskeleton. The cytoskeleton acts as a mechanosensor through the intercalated discs, microtubules, and other cytoskeletal structures that detect changes in cell shape or stiffness and lead to changes in the localization and activity of the Hippo pathway main effector proteins, YAP/TAZ, leading to proliferation [75,76,77].

An important aspect of cardiomyocyte cell cycle re-entry is that cycle progression does not always equate to proliferation, as multinucleation due to cytokinesis failure is common in dividing CM. In fact, the majority of human CMs that undergo DNA replication during the postnatal stage do so without completing cytokinesis, resulting in polyploid or multinucleated cells [78,79,80,81,82,83]. Even when CMs are successfully manipulated through pathways like Hippo to enter a proliferative state, prompting them to complete both karyokinesis and cytokinesis remains a major challenge [74,75,84,85,86,87].

Progress has been made towards this goal. For instance, studies have shown that certain transcription factors like E2F/Rb and proteins involved in cytokinesis, such as *Ect2*, determine if CMs become binucleated and lose their ability to divide [88,89,90]. A more targeted approach can help overcome these barriers. The transition overexpression of reprogramming factors such as *Oct4*, *Sox2*, *Klf4*, and *c-Myc* (known together as OSKM) has triggered CMs dedifferentiation and proliferation in murine models, even in binucleated cells [89]. These effects were more significant in neonatal and juvenile CMs but still worked in adult CMs under controlled conditions. OSKM-mediated reprogramming has been shown to recover left ventricular function and reduce scarring after myocardial infarction but also to increment the risks of teratoma formation or other off-target effects if the process was not controlled properly [89].

### 3.1. Signaling Pathways Regulating Cardiomyocyte Proliferation

Cardiomyocyte dedifferentiation involves a strictly controlled breakdown and reorganization of the cytoskeletal structure by molecular signaling pathways, while also suppressing others that block cell cycle progression [10,91]. One of the most well-studied regulatory pathways of CM proliferation is the Hippo pathway. When active, this pathway prevents cell proliferation by phosphorylation, retention in the cytoplasm, and degradation of YAP and TAZ. Active Hippo prevents YAP from binding with the transcriptional enhanced associate domain (TEAD) in the nucleus, blocking the expression of proliferative genes [92]. The importance of YAP has been shown in several studies that demonstrate how induced YAP promotes cardiac repair, while knockouts lead to embryonic lethality [75,93]. Moreover, YAP activation has been linked to the cytoskeleton by the upregulation of genes encoding for proteins such as spectrin and calpain (*Sptan1* and *Capn2*, respectively) involved in sarcomere breakdown and turnover [10]. Furthermore, YAP also strengthens vinculin-mediated-cell-to-cell-adhesions and reduces ECM tension, facilitating mitotic rounding [10,74].

Besides Hippo, another important player in CM proliferation is the Wnt signaling pathway. This pathway regulates microtubule network stability and has been proved to rescue microtubule depolymerization caused by colchicine, a tubulin-destabilizing drug [94]. Wnt can regulate the inhibition of glycogen synthase kinase 3B (GSK-3B), which orchestrates tubulin network dynamics [95].

As briefly mentioned in the previous section, the growth factor neuregulin-1 (NRG1) controls CM proliferation by activating the ErbB family of tyrosine kinase receptors [72]. It is important to note that this relationship is heavily dependent on ERBB2, a co-receptor of NRG1, whose expression is inversely proportional to CMs maturation. ERBB2 is required to allow NRG1-driven proliferation and sarcomere breakdown in neonatal hearts [72]. Temporary activation of ERBB2 after birth can allow proliferation in juvenile and adult CMs by promoting cytoskeletal remodeling, YAP activation, and gene expression changes, all characteristics that appear to last long enough to protect against cardiac injury [72,96,97].

### 3.2. Cell Cycle Progression and Cardiomyocyte Proliferation

Cell cycle re-entry is only one step out of many to successfully achieve CM proliferation. Even if CMs are able to enter the cycle after a stagnant period, cell division remains inefficient since most cells arrest before cytokinesis and produce polynucleated CMs [98].

To address these issues, several studies have manipulated cell cycle regulators. For instance, overexpression of cyclin-dependent kinases (CDK1 and CDK4) along with cyclins B1 and D1 in mouse, rat, and human cells induces DNA synthesis, karyokinesis, and successful cytokinesis in some cases, both in vivo and in vitro [80,85,99,100]. However, the proliferative response of CMs is limited, since there are proteasome-mediated mechanisms that help reduce the risk of uncontrolled proliferation [85]. Combining the expression of these cell cycle factors with pharmacological inhibitors of Wee1 and TGFβ enhances progression through the G1/S and G2/M phases, improving CM proliferation efficiency [85,101].

In addition, structural and metabolic remodeling are necessary to drive CM division. Sarcomere disassembly and metabolic changes during mitosis are required for proper cytokinesis. Several endogenous transcriptional regulators also influence CM proliferation. Meis1, a homeodomain transcription factor, increases in expression during the early postnatal period as CMs withdraw from the cell cycle [102]. It contributes to arrest by activating CDK inhibitors like *p15*, *p16*, and *p21*, which suppress cell cycle progression and promote maturation [102]. *Meis1* deletion extends the time frame CMs can proliferate and can even induce mitosis in adult hearts without impairing function [102].

*Hoxb13*, another transcription factor with a similar expression timeline, works in tandem with Meis1 to arrest the cycle [103]. Deleting both *Hoxb13* and *Meis1* improves cardiac function after injury by promoting CM mitosis and sarcomere disassembly [102,103]. *Hoxb13* has also been linked to calcium signaling, specifically via calcineurin-dependent dephosphorylation [103].

Collectively, these findings emphasize that cell cycle progression by itself is insufficient for division; equally important are transcriptional, structural, and metabolic factors that contribute to successful proliferation.

### 3.3. MicroRNAs in Cardiomyocyte Proliferation

MicroRNAs (miRNAs) play a central role in CM proliferation and cardiac repair. These small, non-coding RNAs regulate gene expression post-transcriptionally by binding to complementary mRNAs, either blocking their translation or marking them for degradation. In the heart, miRNA disruption has been linked to cardiomyopathy, heart failure, and impaired recovery after injury [104,105,106,107].

Some miRNAs, like miR-1, miR-133, and members of the miR-15 family, suppress proliferation. Others, including miR-590 and miR-199a, have been shown to promote proliferation in neonatal and adult CMs, both in vitro and in vivo [105]. These miRNAs target genes that block cell cycle progression, such as components of the Hippo pathway (TAOK1 and β-TrCP), and actin cytoskeleton regulators like *Cofilin2* [108,109,110]. For instance, studies have shown that delivering these miRNAs to mouse hearts after myocardial infarction promoted cell division, reduced scar formation, and restored function [105,108]. Remarkably, these effects were also observed in fully mature CMs, once thought incapable of re-entering the cell cycle.

These findings led researchers to test miRNAs in large animal models. Delivering miR-199a to pigs via adeno-associated virus significantly improved cardiac function, increased muscle mass, and reduced scar tissue within weeks [108]. However, serious challenges remain. Despite initial improvement, most pigs died suddenly from arrhythmias within two months [108]. Ion channel protein levels remained unchanged, suggesting the arrhythmias were not due to electrical remodeling. The likely cause was structural instability from accumulating immature CMs.

miRNAs are powerful tools for cardiac repair, but they carry significant risks. While miR-590 and miR-199a indicate that CM division can be stimulated, tight control over timing, dosage, and delivery is essential to avoid life-threatening consequences.

### 3.4. Metabolic Shifts and Cytoskeletal Remodeling in Proliferating Cardiomyocytes

How CMs generate energy strongly influences whether they continue dividing or start maturing. Early in development, CMs rely on glycolysis. After birth, they switch to oxidative phosphorylation (OxPhos), which is more efficient in oxygen-rich conditions [62]. This switch halts proliferation and drives maturation, partly due to increased reactive oxygen species (ROS) that activate stress responses like the DNA damage response (DDR) [61]. Hypoxic conditions, which produce less ROS, can delay maturation and preserve proliferative potential [61,111]. Additionally, postnatal environmental changes reduce HIF levels, a key glycolysis promoter.

To restore regenerative potential, researchers have tried shifting metabolism back toward glycolysis. Strategies include blocking fatty acid oxidation or inhibiting enzymes like succinate dehydrogenase (SDH) [62]. Deleting regulators like pyruvate dehydrogenase kinase 4 (PDK4) or the rate-limiting enzyme carnitine palmitoyltransferase 1b (CPT1B) prolongs the proliferative window by pushing cells into anaerobic metabolism [2,11].

Glucose availability is also crucial: more glucose supports proliferation by fueling pathways like the pentose phosphate pathway, which provides DNA precursors. However, excess glucose can impair proper maturation [112].

Metabolism shapes gene expression as well. For instance, fluctuations in alpha-ketoglutarate can modulate histone demethylases like KDM5, altering marks such as H3K4me3 [11]. These epigenetic changes affect whether cells mature or proliferate. Succinate, another metabolic byproduct, accumulates during ischemia–reperfusion and causes oxidative DNA damage [113]. Inhibiting SDH with malonate reduces ROS, promotes glycolysis, and restores regenerative capacity [62]. With careful control over ROS levels and metabolic byproducts, regeneration and structural remodeling might be possible [62].

### 3.5. Extracellular Matrix and Cytoskeletal Regulation of Cardiomyocyte Proliferation

In conditions like heart failure and hypertrophy, defects in the cytoskeleton often play a central role. The cytoskeleton also interacts with proliferation pathway effectors and can help coordinate cardiac repair. YAP acetylation, influenced by its interaction with α-tubulin (TUBA4A), can determine its cellular localization and whether repair occurs following injury [9]. Outside the cell, the extracellular matrix provides both structural support and biochemical signals that influence CM behavior. The dystrophin–glycoprotein complex connects the ECM to the cytoskeleton and transmits mechanical signals that impact proliferation [8,12,13]. In newborn hearts, the ECM protein Agrin is abundant and binds Dag1, disrupting the DGC and activating effectors like YAP and ERK from the Hippo and MAPK pathways [8,12]. These events promote cell division. As the heart matures, the ECM becomes stiffer, diminishing the intensity and nature of signals that reach mechanosensitive regulators [114,115].

Ultimately, ECM and cytoskeletal remodeling together shape CM fate and regenerative potential. In the next section, we explore one cytoskeletal component critical to cardiac contraction: the sarcomere.

### 3.6. Sarcomere Disassembly During Cardiomyocyte Proliferation

A major trade-off during CM maturation is losing proliferative capacity in exchange for contractile function [97,116]. Simply put, cardiomyocytes that divide cannot contract effectively, and vice versa [97]. Successful cell division requires sarcomere disassembly—a process regulated by actin-associated proteins like adducin and mechanotransduction pathways that drive mitotic entry [91]. During interphase, actin remodeling is needed to pass the G2 and G2/M checkpoints [91,117].

While the exact mechanisms remain incompletely understood, it is known that transcription factors mRTF-A and mRTF-B respond to changes in stiffness via RhoA and ROCK, which polymerize actin and translocate the mRTFs into the nucleus [118,119,120]. There, they activate SRF, regulating genes involved in cytoskeletal reorganization and proliferation. YAP also contributes to these changes by interacting with the F-actin cytoskeleton at the Z-discs, which define sarcomere boundaries. YAP controls genes involved in F-actin polymerization, ECM-actin connections, and the expression of actin regulators like Cofilin 1/2 and Gelsolin [121,122]. This RhoA–actin–MRTF and Hippo pathway crosstalk helps coordinate CM proliferation and sarcomere remodeling.

The ubiquitin-proteasome system (UPS) also influences both proliferation and sarcomere turnover. TRIM family E3 ligases, including MuRF1, MuRF2, and MuRF3, target key sarcomeric proteins like Myosin heavy chain, TITIN, and Troponin I [123,124,125]. Fbxo28 (Atrogin 1) contributes to sarcomere breakdown by degrading actin, tropomyosin, troponins, and Z-band components in the same pathway [126,127]. Conversely, UPS proteins like MYBPC3 promote sarcomere stability and density. However, MYBPC3 is a ubiquitination target of MuRF1, which reduces microfilament stiffness and lowers sarcomere density [128,129,130].

Other factors, such as inflammatory cytokines, also regulate transient sarcomere states. Oncostatin M (OSM), part of the IL-6 family, promotes CM dedifferentiation and induces sarcomere degradation by mediating MMP2, SDF1, and VEGF activity [71].

Although OSM can induce both dedifferentiation and degradation, the reversibility of this process remains unclear. Some studies suggest that short-term OSM exposure allows recovery, while prolonged activation impairs contractility [71]. In parallel, NLRP3 inflammasome activation, with subsequent IL-1β/IL-18 releases and pyroptosis, plays a major role in cardiac and vascular disease progression. Both experimental and early clinical studies show that NLRP3 inhibitors (e.g., colchicine, OLT1177, INF4E) can reduce infarct size, improve mitochondrial function, and prevent adverse remodeling after ischemic injury [131,132]. However, how these inflammatory signaling regulate cytoskeletal structures during CM dedifferentiation and proliferation require further investigations.

NRG1 is another reprogramming signal that acts via ERBB4 receptor [73]. NRG1 promotes cell division, actin cytoskeleton remodeling, and mitotic spindle orientation while coordinating motility and proliferation [133]. Overexpression of ERBB2 promotes an epithelial–mesenchymal transition (EMT)-like regenerative response characterized by cytoskeletal remodeling, junctional dissolution, increased cell migration, and extracellular matrix turnover. Notably, YAP is activated and functions downstream of ERBB2 through a signaling circuit that involves cytoskeletal and nuclear envelope remodeling, as well as ERK-dependent mitogenic phosphorylation of YAP at S274 and S352 [96].

These mechanisms highlight the critical role of mechanical signaling in sarcomere remodeling. Adhesive signaling also contributes substrates that are either too soft or too stiff inhibit sarcomere assembly in neonatal CMs, promoting growth and division [134]. In contrast, intermediate stiffness and proper N-cadherin alignment facilitate sarcomere maturation and induce cell cycle exit.

## 4. Conclusions

Cardiomyocyte proliferation is a complex process that depends on carefully coordinated changes inside and outside the cell. To re-enter the cell cycle, cardiomyocytes have to remodel their cytoskeleton, shift their metabolic state, and interact dynamically with their surroundings [135]. During heart development, the cytoskeleton evolves from a flexible, adaptable structure that supports growth into a highly organized, contractile network designed for the constant demands of heart function. But that same specialization comes at a cost: it limits the heart’s ability to regenerate.

Key signaling pathways, especially the Hippo pathway and its effector YAP, help translate mechanical signals from the cytoskeleton and extracellular matrix into the molecular programs that drive dedifferentiation and cell cycle re-entry. At the same time, metabolic reprogramming—from oxidative phosphorylation back to glycolysis—helps meet the energy needs of proliferating cardiomyocytes, while epigenetic changes reset gene expression to support this shift. Even the extracellular matrix, through structures like the dystrophin–glycoprotein complex, plays an active role in guiding these changes by shaping cytoskeletal dynamics and YAP activity. We have made exciting progress in understanding how these components fit together, but much of the picture is still missing. How exactly these signaling pathways, metabolic shifts, and mechanical forces converge to allow true, functional proliferation remains an open question. Nevertheless, this growing body of knowledge brings us closer to the goal of regenerating heart tissue after injury. Approaches such as targeting YAP signaling, reprogramming metabolism, or remodeling the extracellular environment hold promise for reactivating cardiomyocyte proliferation. However, inducing proliferation is only part of the challenge. Real cardiac regeneration depends not just on making new cells, but on making sure those cells mature, reorganize their sarcomeres, and reconnect electrically with the rest of the heart. Without that final step, new cells may fail to support contraction or disrupt normal heart function. Recent studies have made this clear. Sustained activation of proliferative pathways like Hippo/YAP promotes cardiomyocyte cell-cycle reentry, but without subsequent re-differentiation, myocardial architecture is disrupted and contractile function declines. The path forward is about finding the right balance and timing stimulating proliferation while ensuring the re-maturation and integration of newly formed cardiomyocytes into the functional myocardium.

## Data Availability

No new data were created or analyzed in this study.
